# Cross View Gait Recognition Using Joint-Direct Linear Discriminant Analysis

**DOI:** 10.3390/s17010006

**Published:** 2016-12-22

**Authors:** Jose Portillo-Portillo, Roberto Leyva, Victor Sanchez, Gabriel Sanchez-Perez, Hector Perez-Meana, Jesus Olivares-Mercado, Karina Toscano-Medina, Mariko Nakano-Miyatake

**Affiliations:** 1 Instituto Politécnico Nacional, ESIME Culhuacan, 04430 Coyoacán, CDMX, Mexico; jportillop1300@alumno.ipn.mx (J.P.-P.); gasanchezp@ipn.mx (G.S.-P.); jolivares@ipn.mx (J.O.-M.); ltoscanom@ipn.mx (K.T.-M.); mnakano@ipn.mx (M.N.-M.); 2 Department of Computer Science, University of Warwick, CV4 7AL Coventry, UK; M.R.Leyva-Fernandez@warwick.ac.uk (R.L.); V.F.Sanchez-Silva@warwick.ac.uk (V.S.)

**Keywords:** gait recognition, view-invariant methods, gait energy image (GEI), direct linear discriminant analysis (DLDA), KNN classifier

## Abstract

This paper proposes a view-invariant gait recognition framework that employs a unique view invariant model that profits from the dimensionality reduction provided by Direct Linear Discriminant Analysis (DLDA). The framework, which employs gait energy images (GEIs), creates a single joint model that accurately classifies GEIs captured at different angles. Moreover, the proposed framework also helps to reduce the under-sampling problem (USP) that usually appears when the number of training samples is much smaller than the dimension of the feature space. Evaluation experiments compare the proposed framework’s computational complexity and recognition accuracy against those of other view-invariant methods. Results show improvements in both computational complexity and recognition accuracy.

## 1. Introduction

During the past two decades, the use of biometrics for person identification has been a topic of active research [1]. Several schemes have been proposed by using fingerprints, face iris, retina and speech features, all of which can provide a fairly good performance in several practical applications [2,3,4,5,6,7,8,9,10,11]. However, performance significantly degrades when they operate in an un-constrained environment. Because there are practical applications that operate in un-constrained environments, several biometrics have been developed to carry out person identification in these environments. Among them, gait recognition has received considerable attention [8,9]. Particularly, those gait recognition methods that do not depend on human walking models [12], has been shown to significantly increase accuracy and reduced computational complexity by using information extracted from simple silhouettes of moving persons [13]. In general, several aspects may degrade the performance of gait recognition methods, e.g., clothes, shoes, carried objects, the walk surface, time elapsed, and view angles. Among them, the view angle, which corresponds to the angle between the optical axis of the capturing camera and the walking direction [14], is an important factor because the accurate performance of most appearance-based approaches strongly depends on a fixed view angle [15].

Gait recognition approaches aimed at solving problems related to varying view angles can be classified as (a) view invariant approaches; (b) visual hull-based approaches; and (c) view transformation-based approaches. View-invariant approaches transform samples of different views into a common space; while visual hull-based approaches depend on 3-D gait information, and thus usually require the acquisition of sequences by multiple calibrated video cameras. Bodor et al. [11] propose application of images on a 3-D visual hull model to automatically reconstruct gait features. Zhang et al. [16] propose a view-independent gait recognition algorithm using Bayesian rules and a 3-D linear model, while Zhao et al. [17] propose an array of multiple cameras to capture a set of video sequences that are used to reconstruct a 3-D gait model. These methods perform well for fully controlled and cooperative multi-camera environments; however, their computational cost is usually high [13].

The idea behind view transformation approaches is to transform the features vectors from one domain to another by estimating the relationship between the two domains. These transformed virtual features are then used for recognition [18]. View transformation approaches do not require synchronization of gait data of multiple views of the target subjects. Therefore, these approaches are suitable for cases where the views available in the gallery and probe sets are different [18]. These approaches may employ singular value decomposition (SVD), e.g., [14] or regression algorithms for the matrix factorization process during the training stage [19]. The principal limitation of these approaches is that the number of available images is limited to a discrete set of training views and recognition accuracy degrades when the target view and the views used for training are significantly different.

View-invariant gait recognition approaches can be classified further into geometry-based approaches [20], subspace learning-based approaches [21] and metric learning-based approaches [18]. In geometry-based approaches, the geometrical properties of gait images are used as features to carry out recognition. Using this approach, Kale et al. proposed to synthetize side-view gait images using any arbitrary view. This assumes that the person is represented as a planar object on a sagittal plane [22]. Their method performs well when the angle between the image and sagittal planes of the person is small; however, accuracy is significantly degraded when this angle is large [23]. Subspace and metric learning-based approaches do not depend on this angle. Metric learning approaches estimate a weighting vector that sets the relevance of a matching score related to each feature and uses the weighting vector to estimate a final recognition score [23]. The pairwise RankSVM [24] is used by Kusakunniran et al. [19] to improve gait recognition performance for view angle variation, and for cases when the person wears extra clothing accessories and carries objects.

Subspace learning-based approaches project features onto a subspace that is learned from training data and then estimate a set of view-invariant features. Liu et al. [25] propose an uncorrelated discriminant simplex analysis method to estimate the feature subspace, while Liu et al. [18] propose the use of the joint principal component analysis (JPCA) to estimate the joint gait feature pairs subspace with several different view angles. View-invariant gait recognition methods based on subspace learning approaches have been shown to achieve high recognition rates.

Dimensionality reduction is considered as a within-class multimodality problem if each class can be classified into several clusters [26]. In this case, during the training stage, the system creates a set of clusters using similarities among view angles. To analyze subspaces obtained after dimensionality reduction, a preprocessing step is used to manipulate the high-dimensional data. This is especially important when gait energy images (GEIs) are used as features because the dimensionality of the feature space is usually much larger than the training set. This problem is known as the small sample size (SSS) [27] or under-sampling (USP) problem [28], and results into a singular sample scatter matrix. A common solution for this problem is to use principal component analysis (PCA) [28] for dimensionality reduction of the feature space. A potential problem of this approach is the fact that PCA may discard dimensions containing important discriminant information [29]. In other approaches, such as those in Mansur et al. [30], a model for each view angle (MvDA) is constructed independently; however this approach results in a higher computational cost and requires the use of cross-data set information.

This paper presents an appearance-based gait recognition framework that helps overcome the limitations associated with different view angles. This paper extends our work in [31] by providing a more detailed description of the methodologies, as well as an extensive analysis and comparisons of the framework’s performace. The proposed framework, which is based on subspace learning, employs GEIs as the features. It uses direct linear discriminant analysis (DLDA) to create a single projection model used for classification. This approach differs from previously proposed approaches, like the View Transformation Model (VTM), cross-view and multi-view gait recognition scheme, proposed by Kusakunniran et al. [19], which is based on a view transformation model using multilayer perceptron and reduces the GEIs size. The advantages of the proposed framework, called Joint-DLDA hereinafter are manifold: (1) it does not require creating independent projection models, one for each distinct view angle, for classification. This is particularly useful in practical situations where the test data may be acquired at a view angle that does not exist in the gallery data. A unique projection model for classification of several angles can handle this situation; (2) It can handle high-dimensional feature spaces; (3) It has a considerably lower computational complexity than other approaches, as it uses a simple classification stage. Evaluation performance using the CASIA-B gait database [30] shows that the proposed framework outperforms several recently proposed view-invariant approaches, in terms of recognition rate and computational time.

The rest of the paper is organized as follows. In Section 2, we describe the proposed framework in detail; Section 3 provides the evaluation results; We conclude this paper in Section 4.

## 2. Proposed Framework

**Figure 1:**
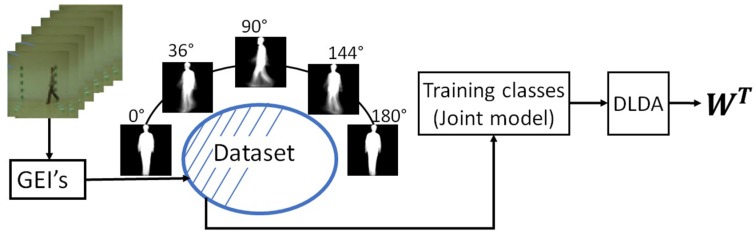
Proposed scheme for constructing the unique projection model.

The proposed gait recognition framework consists of three stages: computation of GEIs, joint model estimation, subspace learning using DLDA and person recognition, as shown in Figure 1. A detailed description of each of these stages is described next.

### 2.1. Computation of GEIs

**Figure 2:**
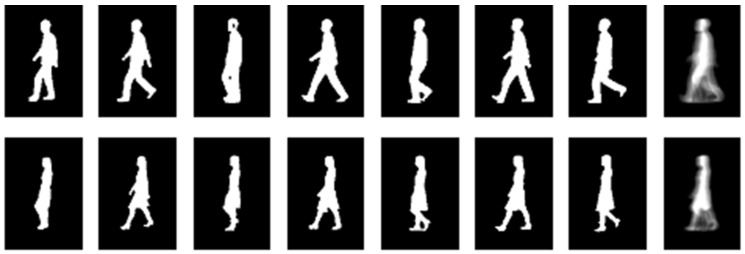
Examples of Gait energy image, last column, computed by using a set of normalized binary sihouette images representing a walking cycle.

Several approaches have been developed for gait representation. A suitable approach is the spatio-temporal gait representation, called gait energy image (GEI), proposed by Han and Bhanu [13], which extracts the human silhouettes of a walking sequence. Then, the extracted binary silhouettes are preprocessed to normalize them such that each silhouette image has the same height and their upper half is centered with respect to a horizontal centroid [13]. A GEI is obtained as an average of the normalized binary silhouettes, as follows [32,33]:

(1) $$G_{j,k,v}\left(x,y\right)=\frac{1}{N_{F}}\sum_{t=1}^{N_{F}}B_{j,k,v,t}\left(x,y\right),\;k=1,2,\cdots ,K,\;j=1,2,\cdots ,J,\;v=1,2,\cdots ,V$$

where $G_{j,k,v}\left(x,y\right)$ is the (x,y)-th gray value of the GEI of *j*-th sequence captured at the *v*-th view angle, which corresponds to the *k*-th class; $B_{j,k,v,t}\left(x,y\right)$ is the (x,y)-th value of the binary silhouette of the *t*-th frame of the sequence; *K*, *J* and *V* are number of classes (persons), sequences per class and view angles per sequence, respectively; and $N_{F}$ is the total number of frames in the walking cycle. Figure 2 shows a set of normalized binary silhouette images representing a walking cycle of two different persons, and the corresponding GEIs.

### 2.2. Joint Model Estimation

**Figure 3:**
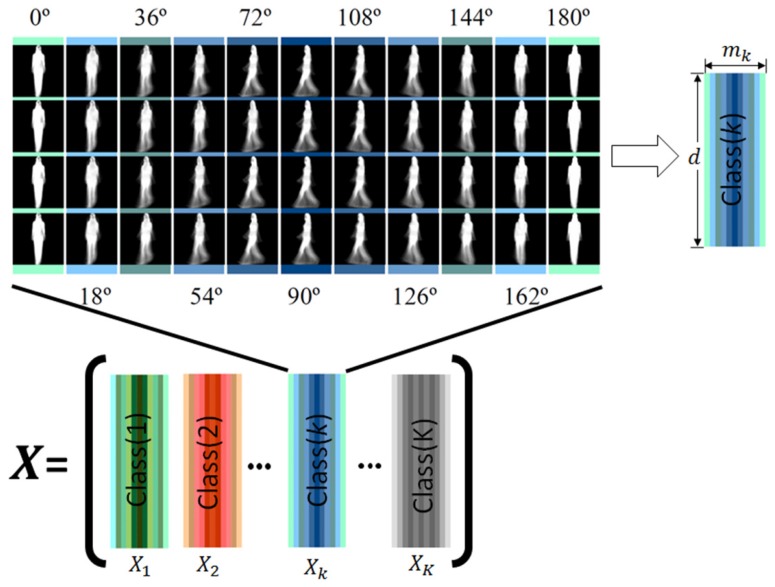
Illustration of the joint model constructed by using the training data corresponding to K-classes using gait energy images (GEIs) of the CASIA-B database [15]. The class (k) in this figure consists of all different view angles V and samples available for subject k.

The proposed framework estimates a joint projection model that avoids creating a model independently for each view angle. Once GEIs of all sequences with different view angles for each person *k* are obtained by Equation (1), these GEIs are concatenated to generate the *k*-th input matrix $X_{k}$, which has a size of $d\times m_{k}$ where *d* is the total number of pixels in each GEI and the size of $m_{k}=J\times V$, where *J* is number of sequences per class and *V* is number of angles per class. The training set X is generated by concatenating all input matrices $X_{k}$, k=1,2,⋯,K, where *K* is the number of classes. The size of the training set X is therefore d×M, where *M* is the total number of GEIs of all classes. Figure 3 shows the generation of training set X.

Since the size of X is too large, a dimensionality reduction method must be used. DLDA is a suitable approach, because it is effective in separating classes and reducing the intra-class variance, while reducing the dimensionality. The discriminant properties of the DLDA ensure that the classes defined by different view angles can be discriminated well enough. In other words, when the training set contains several view angles, the discriminant properties of DLDA can effectively separate the classes represented by the different view angles in the projected subspace; thus allowing for the characterization of query view angles even if they are not included in the training set [8]. Thus, the DLDA is used for estimating a joint projection matrix W from the input matrix X.

### 2.3. Direct Linear Discriminant Analysis

To estimate the joint projection model, consider matrix **X** of size d×M where the samples are stored as *M* *d*-dimensional column vectors that correspond to all possible view angles of all individuals contained in the training set (see Figure 3). Let the number of GEIs in the class *k* be given by $m_{k}$, where $M=\sum_{k=1}^{K}m_{k}$ denotes the total number of GEIs in X; then the matrix $X_{k}\in R^{d\times m_{k}}$ that contains all GEIs belonging to the k-class is given by:

(2) $$X_{k}\left(d,m_{k}\right)=G_{j,k,v}\left(x,y\right)\;d=nx\times ny,$$

where *d* is the number of pixels or features. Then, the matrix containing all GEIs is given by:

(3) $$X=\left[X_{1}|X_{2}|X_{k}\left|\cdots \right|X_{K}\right]$$

**Figure 4:**
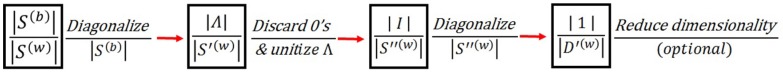
Block diagram of Direct Linear Discriminant Analysis (DLDA).

Next, we employ the DLDA for dimensionality reduction, as shown in Figure 4, to project X into a lower dimensional embedding subspace. Let $z_{i}\in R^{r}:1\leq r\leq d$ be a low-dimensional representation of $X_{i}$, where *r* is the dimension of the embedding subspace while the embedded samples $z_{i}$ are then given by $z_{i}=W^{T}X_{i}$ where $W^{T}$ denotes the transpose of the transformation matrix [29,31].

The purpose of the DLDA is to find a projection matrix W that maximizes the ratio between class scatter matrix, $S^{\left(b\right)}$, and the within-class scatter matrix, $S^{\left(w\right)}$; also known as Fisher’s criterion:

(4) $$\underset{W\in R^{d\times r}}{\arg\;\max}\frac{|W^{T}S^{\left(b\right)}W|}{|W^{T}S^{\left(w\right)}W|}$$

using the procedure described in the block diagram of Figure 4, where:

(5) $$S^{\left(b\right)}=\sum_{k=0}^{K}m_{k}\left(\mu_{k}-\mu \right){\left(\mu_{k}-\mu \right)}^{T},$$

(6) $$S^{\left(w\right)}=\sum_{k=0}^{K}m_{k}\left(X_{k}-\mu_{k}\right){\left(X_{k}-\mu_{k}\right)}^{T},$$

where $\mu_{k}$ is the sample mean belonging to the class *k* and *μ* is the mean of all samples in the dataset. If the number of samples is smaller than their dimension, both $S^{\left(b\right)}$ and $S^{\left(w\right)}$ may become singular. For example, the within-class scatter matrix $S^{\left(w\right)}$ may become singular if the size of the samples is much larger than the number of samples in each class because its rank is at most M−K, this is a common situation in gait recognition applications, as well as some face recognition applications. In order to prevent $S^{\left(w\right)}$ from becoming singular, Belhumeur et al. [10] propose reducing the dimensionality of the features space by using the PCA, such that the pixel or features should be at least equal to M−K, and then applying Linear Discriminant Analysis (LDA), also known as the Fishers criterion, as given by Equation (4). Thus maximizing Fisher’s criterion requires reducing the within-class scatter matrix $S^{\left(w\right)}$ and then incrementing the between-class scatter matrix. Dimensionality reduction by using PCA is based on data variability; PCA allows discarding those dimensions that do not contain important discriminant information. In DLDA, the diagonalization of the between-class scatter matrix $S^{\left(b\right)}$ is given by (see Figure 4):

(7) $$\Lambda =V^{T}S^{\left(b\right)}V$$

where V and **Λ** denote the eigenvectors and eigenvalues of matrix $S^{\left(b\right)}$, respectively. Let Y denote a matrix of dimension d×M, where r≪d. The *M* columns in V correspond to the eigenvectors associated with the largest eigenvalues such that:

(8) $$D_{b}=Y^{T}S^{\left(b\right)}Y$$

where the matrix $D_{b}$ of dimension r×r is a submatrix of **Λ**. Next, let

(9) $$D_{b}^{-1/2}D_{b}D_{b}^{-1/2}=D_{b}^{-1/2}Y^{T}S^{\left(b\right)}D_{b}^{-1/2}$$

(10) $$D_{b}^{-1/2}Y^{T}S^{\left(b\right)}YD_{b}^{-1/2}=I$$

defining $Z=YD_{b}^{-1/2}$, from Equation (10) it follows that:

(11) $$Z^{T}S^{\left(b\right)}Z=I$$

Thus Z unitizes $S^{\left(b\right)}$ and reduces the dimensionality from *d* to *r*. Let us now diagonalize matrix $Z^{T}S^{w}Z$ using the PCA as follows

(12) $$U^{T}Z^{T}S^{\left(w\right)}\mathrm{ZU}=D_{w}$$

where $U^{T}U=I$. Defining $A=U^{T}Z^{T}$, Equation (12) becomes:

(13) $${\mathrm{AS}}^{w}A^{T}=D_{w}$$

By multiplying Equation (11) by $U^{T}$ on the left and by U on the right, and by using $A=U^{T}Z^{T}$, it follows that

(14) $${\mathrm{AS}}^{b}A^{T}=I$$

Because A, diagonalizes $S^{\left(w\right)}$, the dimensionally reduced input vector is then given by

(15) $$W^{T}=D_{w}^{-1/2}A$$

(16) $$X^{*}=W^{T}X$$

The expression in Equation (16) is used for project the gallery and during testing.

**Figure 5:**
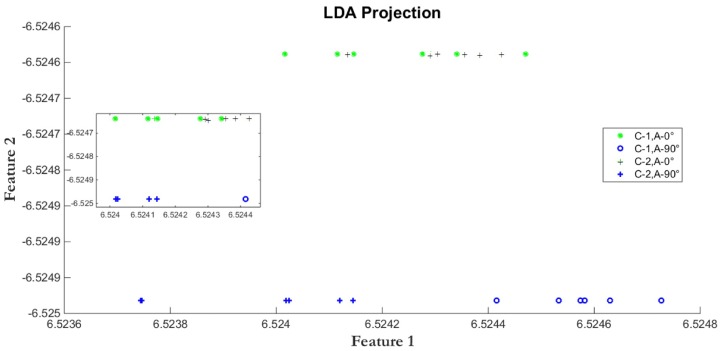
Linear Discriminant Analysis (LDA). The projection is more likely to group the samples according to the view angle rather than according to classes.

**Figure 6:**
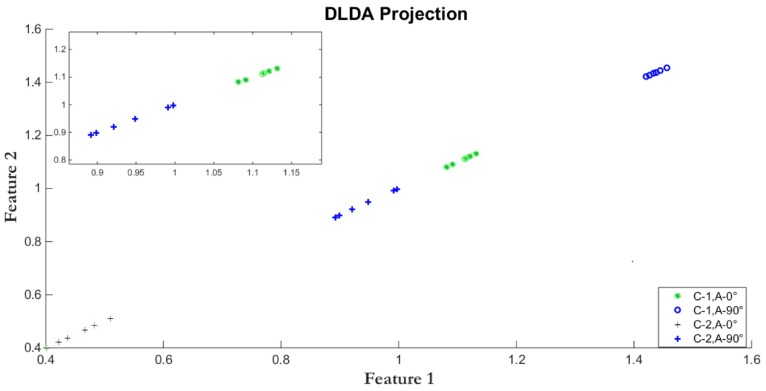
DLDA. The projection is prone to group the samples into classes rather than grouping then according to view angles.

Figure 5 and Figure 6 show the LDA and DLDA projection, respectively, of GEIs belonging to two different classes of the CASIA-B database, where one class is expressed by circles and the other class by crosses. In both classes, two different view angles are used, the $0^{{}^{\circ}}$ view angle is represented by thin circles and thin crosses, in the other hand, the $90^{{}^{\circ}}$ view angle is expressed by thick circles and thick crosses. It is important to note that, even though they might belong to the same class, GEIs from view angle $0^{{}^{\circ}}$ are different from those at $90^{{}^{\circ}}$, thus after projection, clusters of crosses and circles, either thin or thick, should appear. In other words, the projection model must simultaneously reduce the intra-classes variability and to separate the crosses from the circles, which represent a different classes; i.e., increase the distance between classes. Figure 5 shows that LDA tends to cluster the samples according the view angle instead of clustering them by classes; while DLDA (Figure 6) tends to separate the samples by classes instead of view angles, thus allowing to improve the classification even using when using distinct view angles. Thus the projection used must allow for the clustering of all classes independently of the view angle. To achieve this goal, DLDA diagonalizes the scatter matrix $S^{\left(b\right)}$, in order to discard the null space of $S^{\left(b\right)}$ that does not contain useful information instead of discarding the null space of $S^{\left(w\right)}$ that includes the most important information for discrimination purposes [29]. By using DLDA, we obtain a transformation matrix W that projects the data into a low dimensional subspace with an appropriate class separability.

### 2.4. Gallery Estimation

**Figure 7:**
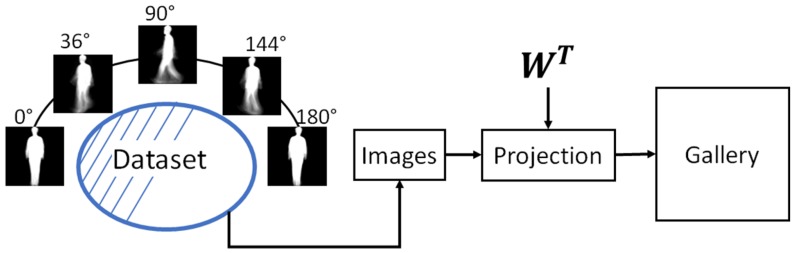
Block diagram of gallery construction.

After the projection model is estimated using DLDA, the gallery of images used by the KNN classification stage is projected as follows:

(17) $$X_{G}\left(s\right)=W^{T}X\left(s\right)$$

where *s* is any of the *J* GEIs corresponding to any of the *K* classes from any of the *V* view angles, available in the gallery set. Figure 7 shows the block diagram of the gallery estimation process.

### 2.5. Classification Stage

During classification, the system uses a GEI of the person to be identified, $X_{PG}$, which is projected into a dimensionally reduced space, using Equation (16), as follows:

(18) $$X_{P}=W^{T}X_{PG}$$

**Figure 8:**
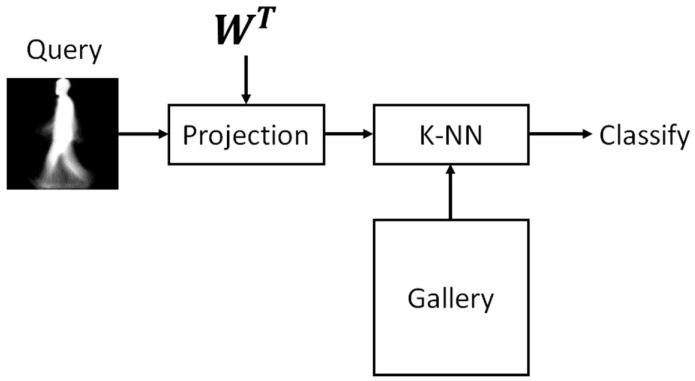
Classification stage.

$X_{P}$ is fed into the KNN stage, where $X_{P}$ is compared with the features vectors $X_{G}\left(s\right)$ stored in the database. Next, the distance between the input vector and those contained in the gallery is estimated, keeping the *K* vectors $X_{G}\left(j\right)$ with the smaller distance. Finally, the class label of the input to which the GEI belongs is the class with the larger number of previously estimated *K* projected vectors. The classification process is illustrated in Figure 8.

## 3. Evaluation Results

Performance of the proposed framework recognition algorithm was evaluated using the CASIA-B gait database [8] with the GEI features obtained using the method proposed by Bashir et al. [8]. The CASIA-B database consists of 124 subjects (classes), each with 11 incoming angles with 10 walking sequences per angle from $0^{{}^{\circ}}$ to $180^{{}^{\circ}}$ with a separation among them of $18^{{}^{\circ}}$. These sequences include six normal walking sequences that are used to perform the experiments. The size of GEIs used in the proposed framework is equal to 240×240.

The proposed framework is evaluated using three different configurations. The first configuration is similar to that proposed by Mansur et al. [30], which is used to evaluate their MvDA method. The second configuration is used to evaluate the VTM models proposed by Kusakunniran et al. [34,35,36], besides the configuration proposed by Bashir et al. [8]. Finally, the recognition performance of the proposed framework is also evaluated using the configuration used by Yu et al. [15], which employs an structure for evaluating the effect of the view angle.

Mansur et al. [30] propose to use two different databases, the CASIA-B and the OULP. For the CASIA-B database, they use two non-overlapping groups. The first one comprises 62 classes and is used for training; the second one comprises the remaining 62 classes and is used for testing. The testing group is divided in two subsets: gallery and probe, where the gallery subset consists of the six samples of each class corresponding to the view angle of $90^{{}^{\circ}}$ available in the testing group; while the probe subset is divided in five subsets containing, each, the six samples corresponding to view angles $0^{{}^{\circ}}$, $18^{{}^{\circ}}$, $36^{{}^{\circ}}$, $54^{{}^{\circ}}$ and $72^{{}^{\circ}}$ of the 62 classes available in the testing group. Mansur et al. [30] also use the database OULP to construct the training set with 956 persons at view angle of $85^{{}^{\circ}}$; while the testing set includes, beside the CASIA-B classes described above, samples of the OULP database with view angles of $55^{{}^{\circ}}$ and $75^{{}^{\circ}}$. Mansur et al. propose to increase the number of samples contained in the database CASIA-B, by rotating the view angle in the CASIA-B to obtain the samples with view angles of $180^{{}^{\circ}}$, $162^{{}^{\circ}}$, $144^{{}^{\circ}}$, $126^{{}^{\circ}}$ and $108^{{}^{\circ}}$. Because the angles in both databases are not the same, the view angles of $85^{{}^{\circ}}$, $55^{{}^{\circ}}$ and $75^{{}^{\circ}}$ of the OULP are added to the view angles contained in the CASIA-B database.

In our experiment, we use only the CASIA-B database, which is divided in two non-overlapping groups: the training group with 62 classes and the testing group with the remaining 62. The gallery subset is build using the six samples of each class at view angle $90^{{}^{\circ}}$, and the probe subset comprises the remaining samples of each class; i.e., the six samples of each class at view angles $0^{{}^{\circ}}$, $18^{{}^{\circ}}$, $36^{{}^{\circ}}$, $54^{{}^{\circ}}$ and $72^{{}^{\circ}}$.

Only for this configuration we employ two transformation matrices called JDLDA(1) and JDLDA(2). The first transformation matrix JDLDA(1), is obtained using only the samples available in the training group, without any modification, to show that the proposed method is able to solve the small sample size problem. The second transformation matrix, JDLDA(2), is obtained when increasing the number of samples in the training group, by rotating the samples of the view angles $180^{{}^{\circ}}$, $162^{{}^{\circ}}$, $144^{{}^{\circ}}$, $126^{{}^{\circ}}$ and $108^{{}^{\circ}}$ of the training group. The evaluation results obtained are shown in Table 1.

The performance obtained using the second configuration described above is compared with the framework proposed by Yu et al. [15], where only the CASIA-B database is used. In this configuration, four samples for each one of the 11 view angles in each one of the 124 classes are used to build the training subset and estimating the projection matrix. This procedure is also followed for the gallery. The remaining two samples for each one of the 11 view angles in each class are used for testing.

**Figure 9:**
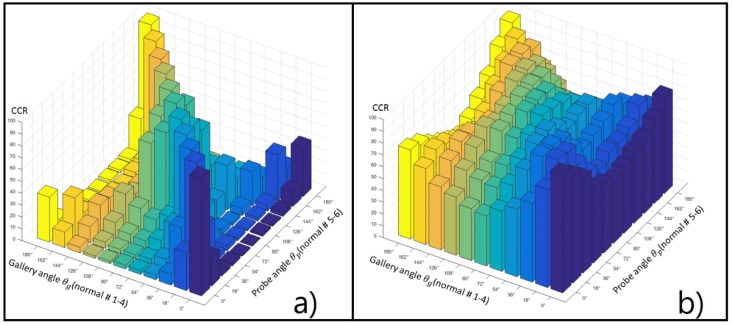
Graphical comparison between the evaluation results provided in [15] showing in (**a**); and those obtained using the proposed framework with the same experimental setup showing in (**b**).

The testing is performed by using all samples available in the gallery subset, fixing each one of the 11 view angles $\theta_{G}$ as gallery, using all samples available in the probe subset by varying each one of the angle $\theta_{P}$, contained in this subset. The evaluation results obtained by [15] are presented in Table 2, where each row corresponds to the results obtained for each view angle, $\theta_{G}$, while each column belongs to a testing view angle $\theta_{P}$. These results are shown in Figure 9a. The evaluation results obtained with the JDLDA are shown in Table 3 and Figure 9b. In this case the transformation matrix is obtained using the training and gallery as proposed by Yu [15].

In the third configuration, only the CASIA-B database is used following two rules to divide the data. In the first rule [19,34,35,36], the database is divided in two groups: The training group, which consists of 24 classes, and the testing, which has the remaining 100 classes. In the second rule [8], the training group consists of 74 classes and the testing group comprises the remaining 50 classes. In both rules, the training and testing groups do not non-overlap and the testing group is divided into the gallery and probe subsets. The gallery subset consists of the four samples of each angle of all classes available in the testing group; while the probe subset consists of the remaining two samples of each view angle of each class of the testing group. To evaluate the performance of proposed framework, all samples of each view angle of the gallery subset are compared with the samples in the probe subset ordered according to the view angle. The results obtained using the rule 1 are shown in Table 4; while the results obtained using the rule 2 are shown in Table 5. In both cases, each row corresponds to the results obtained for a given gallery view angle $\theta_{G}$ while each column belongs to the variation of a given probe view angle $\theta_{P}$.

Table 1, Table 2, Table 3, Table 4 and Table 5 show that the proposed framework provides a very competitive recognition rate. The following are significant features of JDLDA: it does not require the use of two different datasets or modification of the samples size in *X* to overcome the USP; it achieves its best performance for the most challenging angle, i.e., $0^{{}^{\circ}}$ and $180^{{}^{\circ}}$, and finally, it provides very competitive recognition rates when a simple 1-NN classification model is used. The proposed framework, JDLDA(2), achieves a recognition rate close to 100% when the probe view angle is $72^{{}^{\circ}}$ (see Table 1).

Figure 9a shows the graphical comparison between the evaluation results obtained by Yu et al. [15] and those obtained using the proposed framework Figure 9b. In both cases, the same experimental setup is used. Figure 9 shows that the proposed framework provides a higher correct classification rate (CCR) than the system reported in [15] even when the view angle of the gallery data and that of the probe data are different.

**Figure 10:**
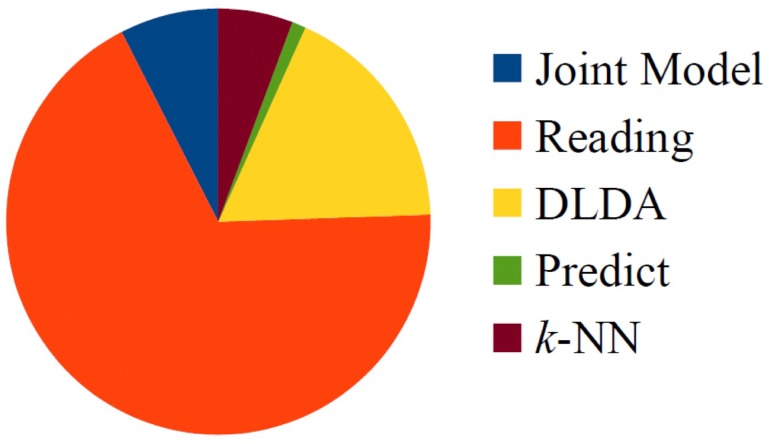
Computation load of proposed framework.

The main drawback of some existing state-of-the-art methods, e.g., VTM and MvDA, is their requirement of building an independent model for each probe view angle to partially overcome the USP. This is an important limitation because these methods imply previous knowledge about the view angles to be tested. The proposed framework does not require any previous knowledge about the probe view angles. Other approaches have been proposed that depend on a single transformation matrix, but they usually require increasing the number of samples to overcome the USP [30]. In these approaches, the view angles of the extra samples and those of the test samples must be close; this situation greatly reduces the ability to transfer the estimated parameters across two different gait datasets if the view angles included in them are not relatively close. Another advantage of our scheme is the time required for classification. Some methods such as that proposed in [14] may require up to 6 h for performing system training [19]. The proposed JDLDA framework is a much more efficient framework not only because it provides a higher recognition rate, but also because it requires as few as 25 s per test. This means that a complete set of experiments may take approximately 40 min. Figure 10 shows the main time-consuming processes in the proposed framework, as a rate of total consumed time. These processes are reading the GEI features from the dataset, creating the joint model, the computing matrix $W^{T}$, generating the k-NN model and classification. From Figure 10 it can be observed that the most time-consuming step is reading the dataset to create the joint model.

## 4. Conclusions

This paper proposed a framework for view-angle invariant gait recognition that is based on the estimation of a single joint model. The proposed framework is capable of classifying GEIs computed from sequences acquired at different view angles. It provides a higher accuracy, with a lower computational complexity than other previously proposed approaches. The estimated joint model used in the framework, which is based on DLDA, helps to reduce the under-sampling problem with remarkable results. Evaluation experiments indicate that it is possible to obtain a projection matrix independently of the gallery subset, which allows us, in several practical applications, to include new classes without the need for recalculating the projection matrix. The evaluation results also show that proposed scheme improves the performance of several previously proposed schemes, although its performance still degrades when the incoming angle and the gallery angle are different. Therefore, in the future it should be interesting to analyze the possibility of developing a gait recognition scheme based on a global model which would be able to keep the same performance independently of the difference between the incoming and gallery angles.

## Figures and Tables

**Table 1:** Recognition performance of several gait recognition algorithms using the CASIA-B database.

Method	0°	18°	36°	54°	72°
**GMLDA [37]**	2%	2%	1%	2%	4%
**DATER [38]**	7%	8%	18%	59%	96%
**CCA [25]**	2%	3%	5%	6%	30%
**VTM [14]**	17%	30%	46%	63%	83%
**MvDA [30]**	17%	27%	36%	64%	95%
**JDLDA(1)**	16%	21%	32%	50%	84%
**JDLDA(2)**	20%	25%	37%	58%	94%

**Table 2:** Evaluation results reported in [15].

		Probe Angle $\theta_{P}$ (Normal Walking #5–6)										
		0°	18°	36°	54°	72°	90°	108°	126°	144°	162°	180°
Gallery angle $\theta_{G}$ (normal #1–4)	**0°**	99.2	31.9	9.3	4.0	3.2	3.2	2.0	2.0	4.8	12.9	37.9
	**18°**	23.8	99.6	39.9	8.9	4.4	3.6	3.6	5.2	13.7	33.5	10.9
	**36°**	4.4	37.9	97.6	29.8	11.7	6.9	8.1	13.3	23.4	13.3	2.0
	**54°**	2.4	3.6	29.0	97.2	23.0	16.5	21.4	29.0	21.4	4.8	1.2
	**72°**	0.8	4.4	7.3	21.8	97.2	81.5	68.1	21.0	5.6	3.6	1.6
	**90°**	0.4	2.4	4.8	17.7	82.3	97.6	82.3	15.3	5.2	3.6	1.2
	**108°**	1.6	1.6	2.0	16.9	71.4	87.9	95.6	37.1	6.0	2.0	2.0
	**126°**	1.2	2.8	6.0	37.5	33.5	22.2	48.0	96.8	26.6	4.4	2.0
	**144°**	3.6	5.2	28.2	18.5	4.4	1.6	3.2	43.1	96.4	5.6	2.8
	**162°**	12.1	39.1	15.7	2.4	1.6	0.8	0.8	2.4	5.2	98.4	28.6
	**180°**	41.1	19.8	8.1	3.2	2.0	0.8	1.6	3.6	12.5	51.2	99.6

**Table 3:** Evaluation results, as presented in [15], but using JDLDA.

		Probe Angle $\theta_{P}$ (Normal Walking #5–6)										
		0°	18°	36°	54°	72°	90°	108°	126°	144°	162°	180°
Gallery angle $\theta_{G}$ (normal #1–4)	**0°**	100.0	92.3	71.4	58.1	52.4	46.8	45.2	52.4	54.4	66.9	81.5
	**18°**	91.1	100.0	98.0	85.9	74.2	61.7	66.9	70.6	68.5	74.2	77.0
	**36°**	82.1	96.8	99.2	97.6	89.1	80.2	78.6	83.5	80.2	76.2	65.7
	**54°**	68.3	83.9	95.6	98.4	94.8	91.9	91.1	86.7	79.0	64.5	54.0
	**72°**	58.1	69.8	87.9	94.4	98.8	98.8	94.8	87.1	69.0	54.4	51.2
	**90°**	50.8	56.5	73.4	86.3	96.4	98.4	98.0	89.9	69.4	53.6	49.2
	**108°**	51.6	59.3	78.2	86.7	95.2	97.6	98.8	97.6	86.7	65.3	52.8
	**126°**	52.4	68.1	81.9	87.9	87.5	89.1	97.6	99.2	96.4	79.0	62.5
	**144°**	62.2	69.0	80.6	84.3	70.6	73.4	89.9	98.0	98.0	89.1	70.6
	**162°**	73.6	79.8	78.2	64.5	60.5	58.5	60.1	83.1	91.5	98.4	88.7
	**180°**	87.8	81.0	66.5	53.2	53.6	45.6	48.0	61.3	72.6	89.9	99.6

**Table 4:** Evaluation results using rule 1 (24 classes for the training group, 100 classes for the testing group, which is divided into gallery subset 1–4 and probe subset 5–6).

		Probe Angle $\theta_{P}$ (Normal Walking #5–6)										
		0°	18°	36°	54°	72°	90°	108°	126°	144°	162°	180°
Gallery angle $\theta_{G}$ (normal #1–4)	**0°**	99.0	43.1	10.5	2.9	1.9	1.7	1.6	2.2	5.4	18.8	39.8
	**18°**	51.7	98.7	63.2	14.7	7.6	4.7	4.6	7.0	14.2	34.3	22.6
	**36°**	19.0	71.4	97.7	57.3	22.1	12.6	12.3	18.8	24.7	24.3	10.1
	**54°**	7.4	17.1	56.1	96.8	43.1	33.2	37.4	37.8	26.4	9.2	3.9
	**72°**	3.2	6.4	18.2	43.0	96.5	76.4	57.2	33.3	12.4	5.4	2.8
	**90°**	1.8	3.9	10.0	31.2	75.3	96.7	87.3	30.7	10.8	3.8	2.2
	**108°**	2.5	4.4	11.0	35.2	58.8	88.0	95.7	61.1	20.7	5.4	2.9
	**126°**	3.8	7.5	21.6	39.1	40.1	35.5	60.5	96.4	70.8	14.6	5.6
	**144°**	8.5	13.1	27.4	26.1	11.2	8.5	19.6	73.3	97.0	25.0	11.4
	**162°**	21.4	36.4	24.5	7.4	4.6	3.6	4.1	9.4	23.7	97.1	51.6
	**180°**	42.6	23.5	9.2	3.4	2.2	2.3	2.9	5.5	12.7	55.8	98.7

**Table 5:** Evaluation results using rule 2 (74 classes for the training group, 50 classes for the testing group, which is divided into gallery subset 1–4 and probe subset 5, 6).

		Probe Angle $\theta_{P}$ (Normal Walking #5–6)										
		0°	18°	36°	54°	72°	90°	108°	126°	144°	162°	180°
Gallery angle $\theta_{G}$ (normal #1–4)	**0°**	99.9	80.9	45.4	21.9	14.7	11.1	10.1	14.0	23.4	45.9	66.4
	**18°**	92.2	100	97.3	62.9	36.6	23.8	24.8	35.5	45.4	63.5	57.1
	**36°**	65.4	97.4	98.8	95.4	73.0	50.8	52.1	61.0	60.5	54.7	38.6
	**54°**	35.0	65.5	94.4	98.7	91.0	82.0	80.3	73.4	61.6	34.1	21.6
	**72°**	19.0	33.4	65.1	88.5	98.8	98.0	90.2	74.4	42.7	22.7	15.6
	**90°**	14.4	19.8	38.4	71.9	97.5	99.1	98.1	74.2	41.1	16.6	12.3
	**108°**	15.5	22.9	45.3	75.1	92.1	98.0	98.7	96.3	72.2	28.6	16.8
	**126°**	23.8	36.7	60.9	71.9	79.0	80.6	95.7	98.5	94.8	61.0	30.4
	**144°**	34.8	48.3	63.0	62.4	48.3	49.0	76.5	95.3	98.7	83.6	49.1
	**162°**	53.4	64.4	52.6	30.9	22.3	19.1	25.3	54.4	80.8	99.0	87.3
	**180°**	73.9	51.0	30.5	14.9	11.7	10.0	11.6	21.0	38.5	83.4	99.7
